# Normalization of Enzyme Expression and Activity Regulating Vitamin A Metabolism Increases RAR-Beta Expression and Reduces Cellular Migration and Proliferation in Diseases Caused by Tuberous Sclerosis Gene Mutations

**DOI:** 10.3389/fonc.2021.644592

**Published:** 2021-06-11

**Authors:** Elhusseiny Mohamed Mahmoud Abdelwahab, Judit Bovari-Biri, Gabor Smuk, Tunde Harko, Janos Fillinger, Judit Moldvay, Vera P. Krymskaya, Judit E. Pongracz

**Affiliations:** ^1^ Departments of Pharmaceutical Biotechnology, University of Pecs, Pecs, Hungary; ^2^ Szentagothai Research Centre, University of Pecs, Pecs, Hungary; ^3^ Department of Pathology, University of Pecs, Pecs, Hungary; ^4^ Department of Pathology, Semmelweis University, Budapest, Hungary; ^5^ Department of Pulmonology, National Koranyi Institute of Pulmonology, Budapest, Hungary; ^6^ Pulmonary, Allergy and Critical Care Division, Department of Medicine, Perelman School of Medicine, University of Pennsylvania, Philadelphia, PA, United States

**Keywords:** tuberous sclerosis gene mutation, RARβ, vitamin A metabolism, retinoic acid, rapamycin

## Abstract

**Background:**

Mutation in a tuberous sclerosis gene (TSC1 or 2) leads to continuous activation of the mammalian target of rapamycin (mTOR). mTOR activation alters cellular including vitamin A metabolism and retinoic acid receptor beta (RARβ) expression. The goal of the present study was to investigate the molecular connection between vitamin A metabolism and TSC mutation. We also aimed to investigate the effect of the FDA approved drug rapamycin and the vitamin A metabolite retinoic acid (RA) in cell lines with TSC mutation.

**Methods:**

Expression and activity of vitamin A associated metabolic enzymes and RARβ were assessed in human kidney angiomyolipoma derived cell lines, primary lymphangioleiomyomatosis (LAM) tissue derived LAM cell lines. RARβ protein levels were also tested in primary LAM lung tissue sections. TaqMan arrays, enzyme activities, qRT-PCRs, immunohistochemistry, immunofluorescent staining, and western blotting were performed and analysed. The functional effects of retinoic acid (RA) and rapamycin were tested in a scratch and a BrDU assay to assess cell migration and proliferation.

**Results:**

Metabolic enzyme arrays revealed a general deregulation of many enzymes involved in vitamin A metabolism including aldehyde dehydrogenases (ALDHs), alcohol dehydrogenases (ADHs) and Cytochrome P450 2E1 (CYP2E1). Furthermore, RARβ downregulation was a characteristic feature of all TSC-deficient cell lines and primary tissues. Combination of the two FDA approved drugs -RA for acute myeloid leukaemia and rapamycin for TSC mutation- normalised ALDH and ADH expression and activity, restored RARβ expression and reduced cellular proliferation and migration.

**Conclusion:**

Deregulation of vitamin A metabolizing enzymes is a feature of TSC mutation. RA can normalize RARβ levels and limit cell migration but does not have a significant effect on proliferation. Based on our data, translational studies could confirm whether combination of RA with reduced dosage of rapamycin would have more beneficial effects to higher dosage of rapamycin monotherapy meanwhile reducing adverse effects of rapamycin for patients with TSC mutation.

## Background

Tuberous sclerosis, angiomyolipoma and lymphangioleiomyomatosis (LAM) are diseases characterised by slow growing tumours that are affecting many parts of the body (1, 2) including the skin, brain, kidneys and the lungs. The above diseases are caused by the mutation of tumour suppressor genes tuberous sclerosis 1 or 2 (TSC or TSC2) (1). The above tumours were initially considered benign, but angiomyolipoma and LAM have recently been reclassified as “low grade, destructive, metastasizing neoplasms” characterised by α-smooth muscle actin (α-SMA), vimentin, desmin and melanoma gp100 (HMB45) markers. Diseases caused by TSC mutations bear all the hallmarks of cancers including genetic mutations, evasion of growth suppression, resistance to cell death, metabolic reprogramming to avoid immune detection, and capability of invasion (3). Loss of TSC activity results in continuous activation of the mTOR pathway, which is also characteristic to various neoplasms where upstream mutations or signalling malfunction both result in mTOR activation (4). mTOR activation alters various cellular functions including cellular proliferation, autophagy, mitochondrial biogenesis, and cellular metabolism. Activation of the mTOR pathway can change anabolic cell growth processes such as protein and lipid synthesis in correlation with external growth factor or nutrient intake (5). In our previous study of LAM, apart from detecting morphological abnormalities in mitochondria and suppression of ROS production, we identified downregulation of the proliferation suppressor nuclear receptors of the retinoic acid gene family both retinoic acid receptor (RAR) and retinoid x receptor (RXR) as well as several miRNA-s that regulate RAR expression including miR29b (6). RAR and RXR are receptors of retinoic acid (RA), a metabolite of vitamin A (7). Both classes of nuclear receptors have three subtypes (α, β, and γ) and in-patient derived LAM cell lines RARβ mRNA expression was found significantly reduced (6). Interestingly, RARβ is the receptor that is associated with the anti-tumour effects of RA (8–10). In many neoplastic diseases, expression of RARβ is often downregulated or lost indicating that RARβ plays an important role in tumour suppression (11). RA is a metabolite of the lipophilic vitamin A (retinol) which is obtained from plant or animal sources in the form of carotenoids and retinyl esters, respectively (12). RA is a lipophilic molecule with three isoforms: all-trans, 9-cis and 13-cis RA and is stored in forms of retinyl esters primarily in the liver as well as the kidneys, lungs and the bone marrow (12, 13). In circulation, retinol is bound to retinol-binding protein (RBP) which enters the cells through RBP receptors (STRA6) (12). In the cells, retinol-dehydrogenase (RDH) or alcohol-dehydrogenase (ADH) oxidize retinol to retinal which is irreversibly converted to RA by the aldehyde dehydrogenase (ALDH) family also known as retinaldehyde dehydrogenase (RALDH) (12). RA binds to cellular retinoic acid-binding protein (CRABP) in the cell that carries RA into the nucleus where it binds to nuclear RARs to function as transcription factors. RA signalling is dependent upon its nuclear availability, controlled among others by RBP1, which is the carrier protein involved in the transport of retinol from the storage site to peripheral tissues (12). Interestingly, the administration of RA not only activates the transcription factor RARβ but also increases its expression (14, 15). As currently the only FDA approved drug to treat angiomyolipoma, tuberous sclerosis or LAM is rapamycin, search for additional therapeutic targets is important. Especially so, as rapamycin can only slow down disease progression and cannot offer a cure. Additionally, rapamycin has significant side effects therefore not all patients can tolerate the treatment (16, 17). Discontinuation of rapamycin, however, leads to rapid disease progression (17). Tissues affected by TSC mutation are low in RARβ expression (18, 19). As, RARβ is regulated by RA (6, 19–21), a product of vitamin A metabolism, we theorised that not just RARβ expression is low in LAM, but it is likely that enzymes of vitamin A metabolism malfunction. To test the theory vitamin A metabolic enzyme expression and activity was tested. Simultaneously, the effect of the metabolic product RA was assessed in cell migration and proliferation alone or in combination with rapamycin using TSC mutant cell lines.

Based on our results, such combination of rapamycin with RA might offer a novel therapeutic strategy if our *in vitro* data could be confirmed in a clinical study.

## Materials and Methods

### Ethical Statement

LAM tissue samples were obtained from lung transplant donors for generation of cell lines, in accordance with the Declaration of Helsinki, approved by the Institutional Review Board at the University of Pennsylvania (22) and provided by the National Disease Research Interchange (NDRI, Philadelphia, PA). LAM patients had given written informed consent and all the collected samples were treated anonymously. Paraffin embedded tissue samples were obtained from the Departments of Pathology at Semmelweis University, Budapest, and from the University of Pecs, Pecs, Hungary and the National Koranyi Institute of Pulmonology, Budapest, Hungary. The study was approved by the Medical Research Council of Hungary (54034-4/2018/EKU).

### LAM Cell Lines, Bronchial Smooth Muscle Cells (BSMC), Normal Human Lung Fibroblast (NHLF) S102 and S103 Cell Lines and Cell Culture Conditions

Primary tissue derived cultures of human LAM cell lines were established in the Department of Medicine, University of Pennsylvania, Pennsylvania, USA (22). Briefly, LAM cells were dissociated from LAM nodules of transplant patients. Each LAM nodule was used to establish individual cell lines (characterized by alpha smooth muscle actin (α***-***SMA) expression, mTORC1 activity, HMB45 immunoreactivity, DNA synthesis, and cell migration) (23). In the current study, four patient-derived individual LAM cell lines were used including LAM-100, LAM-111C, LAM-D9065 and LAM-HUP. As controls, primary cultures of normal, human bronchial smooth muscle cells (BSMC) and normal human lung fibroblasts (NHLF), were purchased from Lonza (Basel, Switzerland). Normal BSMC-s and LAM cell lines were cultured at 37°C, 5% CO_2_ in SMC Growth Medium (insulin, hFGF, GA, FBS and hEGF) (Lonza, Basel, Switzerland). Two angiomyolipoma cell lines were also used in the study and cultured at the above-mentioned conditions. The 621-102 (S102)(TSC2-/-) cell line was generated by introduction of E6/E7 (pLXSN 16E6E7-neo) and human telomerase (pLXSN hTERT-hyg) into a primary culture of TSC2 null human angiomyolipoma cells (24–26). The 621-103 (S103)(TSC2+/+) was generated by stable transfection of TRI102 with wild-type TSC2 (pcDNA3.1 TSC2-zeo) into 621-101 cells (24).

### Haematoxylin Eosin Staining

5 µm thick tissue sections of primary normal and LAM lungs (n=6 each, respectively) were stained in Mayer’s haematoxylin solution (Sigma-Aldrich, St. Louis, USA) for 10 min, washed, then differentiated with 0.25% acetic acid and in eosin solution. Sections were mounted using Vectashield mounting medium (Vector Laboratories, Burlingame, USA). Images were taken using Nikon Eclipse Ti-U inverted microscope.

### Immunofluorescent Staining

Normal, BSMC, NHLF, LAM (four individual cell lines), S103 (TSC2+/+) and S102 (TSC2-/-) cells were cultured for 3 days using Falcon™ chambered cell culture slides (Thermo Fisher Scientific, Waltham, USA). Cell cultures were then fixed with 4% formaldehyde and permeabilized with PBS containing 0.1% Triton-X and 5% BSA. Slides were incubated with primary antibodies (**Table 1**) overnight at 4°C. Slides were washed with TBS for three times then incubated with corresponding secondary antibody (**Table 1**) for 90 min at RT. Nuclei were counter stained with DAPI. Images were acquired using an Olympus IX-81 (OLYMPUS Corporation, Tokyo, Japan) both light and fluorescence microscope.

**Table 1:** Antibodies used in western blot, immunofluorescent staining, and immunohistochemistry.

Antibody	Catalog number	Source	Dilution
Anti-alpha Smooth Muscle Actin	MAB1420	R&D Systems, Minneapolis, USA	10 µg/mL
Anti-mTOR Antibody	Ab25880	Abcam, MA, USA	2 µg/ml
Anti-p70 S6 kinase	Ab32529	Abcam, MA, USA	1:200
Anti-RPS6	Ab12864	Abcam, MA, USA	1:250
Ribosomal Protein S6 Antibody	sc-74459	Santa Cruz Biotechnology	1:100
Anti-RAR beta	ab25880	Abcam, MA, USA	2 µg/ml
Anti-mouse Alexa 488	A28175	Thermo Fisher Scientific, Waltham, USA	1:200
Anti-rabbit Alexa 647	A27040	Thermo Fisher Scientific, Waltham, USA	1:200
Anti-rabbit Alexa 488	A11034	Thermo Fisher Scientific, Waltham, USA	1:200
Anti-mouse Alexa 647	A32728	Thermo Fisher Scientific, Waltham, USA	1:200
Anti- Melanoma gp100 antibody (HMB-45)	Ab787	Abcam, MA, USA	1 µg/ml
anti-RARβ	Ab124701	Abcam, MA, USA	1:100
Goat Anti-Rabbit Immunoglobulins/HRP	P0448	DAKO, Produktionsvej, Denmark	1:50
Goat Anti-Mouse Immunoglobulins/HRP	P0447	DAKO, Produktionsvej, Denmark	1:50

### Immunohistochemistry

5 µm thick tissue sections of primary normal and LAM lungs were stained using immunohistochemistry. First, the slides were rinsed in heated xylene and were washed with a descending series of alcohol to remove paraffin. After deparaffination the slides were rehydrated in distilled water and antigen retrieval was performed by heating the slides in Target Retrieval Solution (pH 6, DAKO, Produktionsvej, Denmark) at 97°C for 20–30 min. Subsequently slides were washed in dH_2_O and endogenous peroxidase activity was blocked with 3% H_2_O_2_ containing TBS (pH 7.4) for 15 min. Then slides were washed three times with TBS containing Tween (0.05%, pH 7.4). Pre-blocking was carried out with 3% BSA in TBS for 20 min before overnight incubation with anti- Melanoma gp100 antibody (HMB-45) (1:100, HMB-45 mouse monoclonal antibody clone: Ab787, ABcam) and anti-RARβ (1:100, anti-RARβ rabbit monoclonal antibody clone: Ab124701, ABcam) primary antibody at 4°C. Following incubation slides were washed with TBS for three times then incubated with peroxidase conjugated secondary antibody (1:100, Polyclonal Goat Anti-Rabbit IgG, DAKO) for 90 min. Antibody labelling was visualized with the help of liquid DAB Substrate Chromogen System (DAKO). For nuclear counterstaining, haematoxylin staining was performed. Finally, slides were mounted with Faramount Aqueous Mounting Medium (DAKO, Produktionsvej, Denmark). Histological evaluation was performed with the help of Panoramic MIDI digital slide scanner (3DHistech, Budapest, Hungary). Image analysis was performed using ImageJ software with IHC toolbox plug-in.

### Rapamycin and Retinoic Acid (RA) Treatments

BSMC, NHLF, LAM (four individual cell lines), S103 (TSC2+/+) and S102 (TSC2-/-) cell cultures were treated with rapamycin and/or RA. The two drugs were used in the following concentrations: 10 or 20 nM rapamycin catalogue: tlrl-rap (InvivoGen, San Diego, USA) and 1 or 2 µM RA (Sigma-Aldrich, St. Louis, USA) for 24h at 37°C, 5% CO_2_.

### Western Blot

Cells were lysed in ice-cold RIPA buffer (Sigma-Aldrich, St. Louis, USA) supplemented with protease inhibitors (Roche Diagnostics, Mannheim, Germany) for 30 min on ice and centrifuged at 16,000 × g for 20 min at 4° C. The supernatant was then used as the cell lysate. The protein content of each cell lysate was assessed using a Qubit protein assay kit (Thermo Scientific, Waltham, MA). 30 µg of total protein was loaded onto Mini Protean gel (Bio-Rad, California, USA), then electrophoresis was followed by overnight blotting onto a nitrocellulose membrane using 10 mA current. The blots then were blocked in 5% non-fat skimmed milk blocking solution (Bio-Rad, California, USA) in TBS-T buffer for 1 h and incubated with primary antibodies (**Table 1**) diluted 1:1000 in 2.5% non-fat skimmed milk powder in TBS-T overnight at 4° C. After washing with TBS-T, the blots were incubated with rabbit anti-goat/HRP diluted in 2.5% non-fat skimmed milk powder in TBS-T for 1 h at room temperature. The immunoreaction was developed with a chemiluminescence HRP substrate and recorded with ImageQuant LAS-4000 imager (GE Healthcare Life Sciences, USA).

### RNA Isolation

Total RNA was extracted from normal BSMC, NHLF and LAM (four individual cell lines) cultures with MN NucleoSpin RNA isolation kit according to the manufacturer’s protocol (Macherey-Nagel, Düren, Germany). The concentration of RNA samples was measured using NanoDrop (Thermo Fisher Scientific, Waltham, USA). Total RNA from human lung tissues were obtained using TRIzol reagent (Invitrogen, Thermo Fisher Scientific, Waltham, USA). RNA (1 µg) was digested with DNase (Sigma-Aldrich, St. Louis, USA) to eliminate any DNA contamination. cDNA was synthesized with high-capacity RNA to cDNA kit (Thermo Fisher Scientific, Waltham, USA). Reverse transcription was performed with random hexamer primers.

### Quantitative qRT-PCR

qRT-PCR was performed using SensiFAST SYBR Green reagent (BioLine, London, UK) in an ABI StepOnePlus system. Gene expressions using sequence specific primers (**S. Table 1**) were analysed with StepOne software and normalized to beta-actin. Changes in gene expression were calculated according to the 2^-ddCt^ method.

### Metabolic Enzyme RT2 Array

cDNA was prepared using RT2 First Strand Kit (Qiagen, Hilden, Germany) according to manufacturers’ protocol using 350ng-1000ng of total RNA as starting material. Metabolic enzymes mRNA expression levels were performed using Human Drug Metabolism: Phase I Enzymes arrays (Qiagen, Hilden, Germany), RT2 SYBR^®^ Green qPCR Mastermix (Qiagen, Hilden, Germany) and results were acquired by Quantstudio 12k flex (Thermo Fisher Scientific, Waltham, USA).

### ALDH and ADH Activity Assay

ALDH Activity Assay Kit (Abcam, MA, USA, ab155893) and Alcohol Dehydrogenase Assay Kit (Abcam, MA, USA, ab102533) were used to test ALDH and ADH activity of LAM and S102 compared to their controls before and after treatments. Activity of cell lysates was assessed using a detection kit and following the manufacturer’s instructions. Enzyme activity induced colour changes were measured at OD450 nm with EnSpire^®^ Multimode Plate Reader (PerkinElmer, Waltham, Massachusetts, USA). Pierce™ BCA Protein Assay Kit (Thermo Fisher Scientific, Waltham, USA) was used to measure protein content and results are presented as the fold change *vs*. control.

### Wound Healing Assay

Cells were grown to 90% confluence in 24 well plates and wound gap was made by scratching the cell with rapamycin (10 nM), RA (2 μM) and rapamycin (10 nM) + RA (2 μM) was after inducing the wound gap. The healing of the wound gap by cell migration and the centre of the gap was monitored with images taking with EVOS light microscopy (Thermo Fisher Scientific, Waltham, USA) and the gap area was quantified using ImageJ software.

### BrdU Click-Ti Proliferation Assay

S103 and S102 cells were cultured using Falcon™ chambered cell culture slides (Thermo Fisher Scientific, Waltham, USA). Proliferation capacity was assessed using Click-iT™ Plus EdU Cell Proliferation Kit for Imaging, Alexa Fluor™ 488 dye (Thermo Fisher Scientific, Waltham, USA). Briefly, cell cultures were treated with rapamycin and/or RA then incubated with EDU solution overnight. Following overnight incubation cells were fixed with 3.7% formaldehyde and permeabilized with PBS containing 0.5% Triton-X. Staining was performed following manufacture instructions using Alexa Fluor^®^ 488 picolyl azide and nuclei were counter stained with Hoechst^®^ 33342. Images were acquired using an Olympus IX-81 (OLYMPUS Corporation, Tokyo, Japan) both light and fluorescence microscope.

### 3D Co-Cultures

3D aggregates were formed as described previously (27, 28). Briefly, normal human lung fibroblasts (NHLF) and bronchial smooth muscle cells (BSMC) were isolated from anonymous donors of different ages and sexes and were purchased from Lonza (Basel, Switzerland). All cells were cultured at 37°C and 5% CO2 in primary cell culture media. NHLF, BSMC and LAM cell types were sub-cultured and mixed at 1:1 ratio then dispensed 3*105 cells/well onto a low-attachment 96-well U-bottom plates (Corning, New York, USA). The 3D aggregate co-cultures were incubated in the presence or absence of 10 nM rapamycin and/or 2 μM RA for 24 h, then collected into cryomold and sectioned for staining.

### Statistical Analysis

Unless otherwise noted, statistical analysis was performed with SPSS version 20 software. S102 and S103 data are presented as mean ± technical error of three replicates and statistical analysis was performed using student t-test. In experiments using primary LAM lung derived cell lines and their controls (an average of BSMC n=4 and NHLF n=4 samples) data are presented as mean ± standard error of mean (SEM), and statistical analysis was performed using the one-way ANOVA. p<0.05 was considered as significant.

## Results

**Figure 1:**
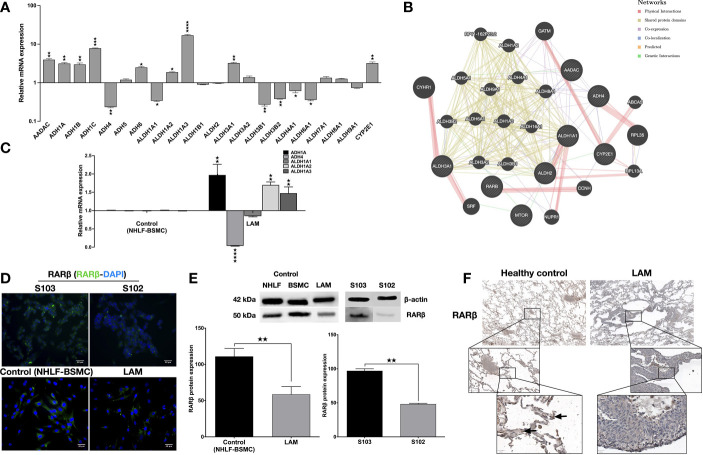
Metabolic enzyme and retinoic acid receptor expression. **(A)** Human metabolic enzyme RT array analysis of S102 (TSC2-/-) compared to S103 (TSC2+/+) control. The figure presentsLog RQ± technical error of n=3 replicates (t-test). **(B)** Predictive analysis of co-expression, physical interactions between metabolic enzymes and RA in TSC mutant diseases groups using GeneMANIA database. **(C)** Gene expression of enzymes involved in vitamin A metabolism measured in LAM cell lines compared to primary healthy controls (BSMC and NHLF, 1:1). Data are presented as mean LogRQ ± SEM compared to untreated control. Significant changes are marked as ★, ★★, ★★★ and ★★★★ (P<0.05, P<0.001, P<0.0002 and P<0.0001, respectively). **(D)** RARβ immunofluorescent staining (RARβ green, nuclei blue, magnification ×40, size bar 28-40 μm). **(E)** Western blot analysis of RARβ protein levels in LAM, control (NHLF and BSMC), S103 and S102 cell lines. WB protein expression levels were quantified by ImageJ and are presented as percentage compared to controls or S103. Significant changes are marked as ★, ★★ and ★★★ (P<0.05, P<0.001 and P<0.0002 respectively). **(F)** RARβ immunohistochemistry of a representative pair of primary LAM lung sections and healthy lung controls (size bar 100-500 μm), (n=6).

**Figure 2:**
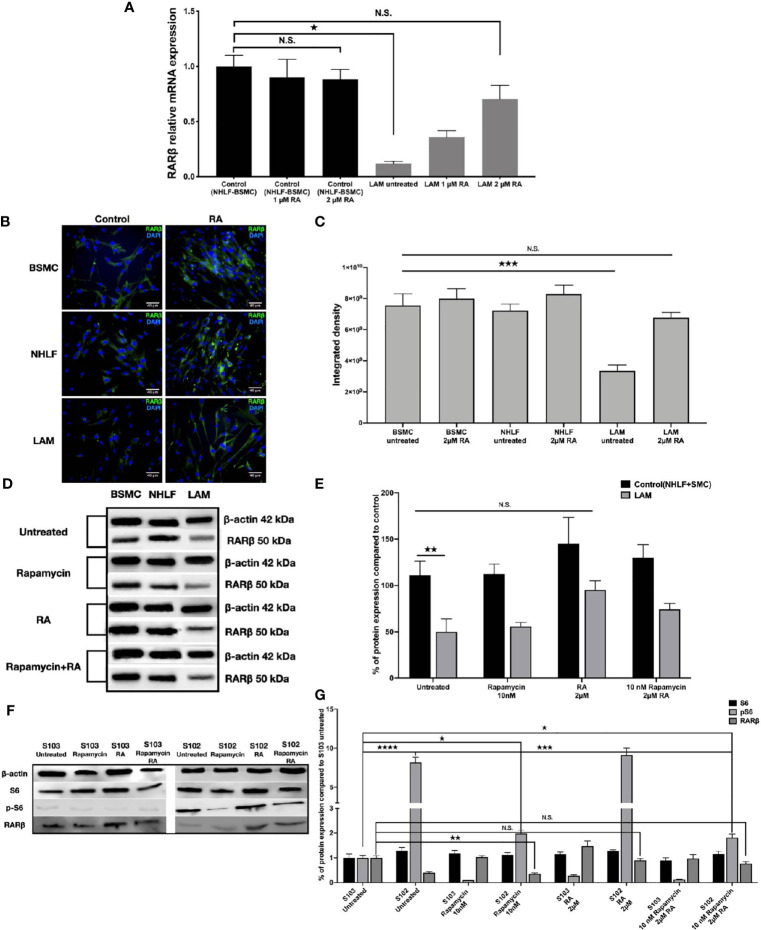
Restoration of RARβ expression by RA. **(A)** mRNA expression levels of RARβ are significantly increased in LAM cell lines (n=4) compared to controls BSMC and NHLF after 2 μM RA treatment for 24 h. Significant changes are marked as ★ (P<0.05). **(B, C),** Protein expression levels of RARβ using immunofluorescence staining in LAM cell lines compared to controls (BSMC and NHLF) after 2 μM RA treatment for 24 h. Immunofluorescence staining RARβ green, DAPI blue, magnification 40x, size-bar 40 μm. **(D, E)** Western blot analysis of RARβ protein levels in LAM cell lines and control cells (NHLF and BSMC). **(F, G)** Western blot analysis of RARβ, S6 and pS6 protein levels in S102 cell line compared to S103. WB protein expression levels were quantified by ImageJ and are presented as percentage compared to controls. Changes are marked as N.S. (Non Significant) or significant ★, ★★, ★★★ and ★★★★ (P<0.05, P<0.001, P<0.0002 and P<0.0001, respectively).

To investigate the involvement of the enzyme cascades associated with vitamin A metabolism in TSC deficient cells, human enzyme profiler arrays (RT2 PCR) were used to compare mRNA levels of specific enzymes in the human kidney angiomyolipoma cell line S102 (TSC2-/-) and its control S103 (TSC2+/+). Out of the alcohol dehydrogenase family, four enzymes (ADH1A, ADH1B, ADH1C and ADH6) were significantly upregulated and one enzyme was downregulated (ADH4) in the mutant cell line (**Figure 1A**). In the aldehyde dehydrogenase family three enzymes (ALDH1A2, ALDH1A3, and ALDH3A1) were upregulated, while five enzymes were downregulated (ALDH1A1, ALDH3B1, ALDH3B2, ALDH4A1 and ALDH5A1) (**Figure 1A**). Additionally, analysis of the array data showed significant increase in CYP2E1 mRNA level (**Figure 1A**, **S. Table 2**). To predict the connection (expression, physical interaction, co-localization, etc) amongst the above described enzymes and RA in TSC mutant cells, a linear regression-based prediction algorithm analysis was performed (GeneMANIA database) (29) (**Figure 1B**, **S. Table 3**). ADHs and ALDHs -especially ADH4, ALDH1A2 and ALDH1A3- were predicted to physically interact with molecules involved in the RA metabolic process and RARβ binding (**Figure 1B**). To determine whether ADHs and ALDHs are present in TSC2-deficient LAM cells, ADHs and ALDHs mRNA expression levels were quantified by qRT-PCR in four patient derived LAM cell lines and normal individual primary human bronchial smooth muscle cell (BSMC) as well as primary normal human lung fibroblast cells (NHLF) as controls (**S. Figure 1**). Just as in the TSC2-/- angiomyolipoma cell line S102, in the primary LAM lung tissue derived cell lines the expression of ADH1, ADH4 and ALDH1A1-2-3 showed the same pattern (**Figure 1C**). Apart from vitamin A metabolism, the importance of ALDH and ADH were demonstrated in cancer cell proliferation, motility and metastasis (30, 31), due to their specific role in affecting mTOR dependent signalling. In a previous study it has been revealed that ALDH1A3 downregulation directly affects mTOR expression and its downstream signals *via* S6K (32). Result that ALDH1A3 mRNA was significantly upregulated in TSC2-/- S102 and patient derived LAM lung cell lines that leads to mTOR activation and downregulation of RARβ expression (6) was confirmed by immunofluorescent staining (**Figure 1D**) and western blotting (**Figure 1E**). To confirm the cell line data, primary normal and LAM lung tissue sections (n=6) were stained for RARβ protein by immunohistochemistry (**Figure 1F**, **S. Figure 2**). The staining of primary LAM tissues confirmed that reduced expression of RARβ expression in the structural cells of the lung tissue is a feature of the TSC mutant LAM lungs (**Figure 1F**).

As RA is known to upregulate RARβ expression (14, 15), we set out to investigate whether RA could restore normal levels of RARβ in TSC2-/- cell lines. Four patient derived LAM lung cell lines were treated with 1 μM or 2 μM (33, 34) RA for 24 h, then RARβ expression was quantified using qRT-PCR (**Figure 2A**) and immunofluorescent staining (**Figures 2B, C**). Following incubation with 2 μM RA, RARβ mRNA (**Figure 2A**) as well as protein expression (**Figures 2B, C**) was restored to normal levels. As patients with LAM disease are treated with rapamycin, and rapamycin is known to downregulate RARβ, RARβ protein expression levels were quantified in patient derived angiomyolipoma and LAM lung cell lines after 10 nM rapamycin treatment in the presence or absence of 2 μM RA (**Figures 2D–G**). While 10 nM rapamycin mono treatment had no effect on RARβ levels, 2 μM RA increased RARβ expression even in combination with rapamycin in the TSC2-/- cell lines (**Figures 2D–G**). The effects of the above treatments were tested on mTOR activity in the angiomyolipoma cell line S102 and its control S103 by western blotting of S6 and pS6 proteins (**Figure 2F**). While 10 nM rapamycin significantly reduced S6 phosphorylation close to control levels (**Figures 2F, G**), pS6 levels in the presence of 2 μM RA mono treatment was not affected and remained just as high as in the untreated TSC-/- control. Combination treatment with 10 nM rapamycin and 2 μM RA resulted in middle ground. Significantly increased but not fully enhanced RARβ protein expression and significantly reduced pS6 levels but not as low as in the presence of 10 nM rapamycin mono treatment (**Figures 2D–G**).

**Figure 3:**
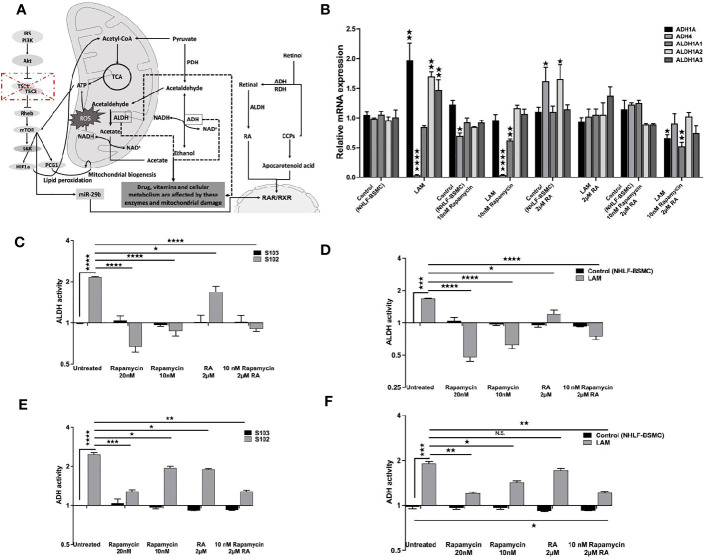
The effect of RA on ALDH and ADH mRNA and enzyme activity levels. **(A)** Schematic figure explaining the connections between different pathways, TSC-mTOR, metabolic enzymes and energy production. **(B)** Gene expression levels of ALDH and ADH enzymes involved in RA metabolism following RA (2 μM) treatment of LAM cell lines compared to controls (BSMC and NHLF). Data are presented as mean LogRQ ± SEM compared to untreated controls. **(C)** ALDH enzyme activity fold changes in S102 (TSC-/-) cell lines compared to untreated S103 (TSC+/+) ± technical error of replicates (t-test). **(D)** ALDH enzyme activity fold changes in LAM cell lines compared to untreated BSMC and NHLF (1:1) ± SEM (ANOVA). **(E)** ADH enzyme activity fold changes in S102 (TSC-/-) cell lines compared to untreated S103 (TSC+/+) ± technical (t-test). **(F)** ADH enzyme activity fold changes in LAM cell lines compared to untreated BSMC and NHLF (1:1) ±  SEM (ANOVA). Changes are marked as N.S. (Non Significant) or significant ★, ★★, ★★★ and ★★★★ (P<0.05, P<0.001, P<0.0002 and P<0.0001, respectively).

Based on the data TSC mutation affects downstream signals including the vitamin A metabolic enzyme signalling cascades (**Figure 3A**). As the FDA approved rapamycin and RA in combination restored RARβ and pS6 levels we also tested both drugs on vitamin A metabolic enzyme expression and activity. Mono treatment with 2 μM RA normalised mRNA expression levels of ADH (1A, 4) and ALDH (1A1, 1A2, 1A3) (**Figure 3B**). Also, in RA treated TSC mutant S102 and primary LAM lung derived cell lines ADH and ALDH enzymes activity showed significant decrease compared to untreated controls (**Figure 3B**). Expression levels (**Figure 3B**) and enzymatic activity (**Figures 3C–F**) of metabolic enzymes were also quantified after 10 and 20 nM rapamycin and/or 2 μM RA treatments. The cultures were assessed after 24 h incubation. While in mono treatment the 20 nM rapamycin was the most efficient in reducing ALDH (35) and ADH activity, combination treatment of 10 nM rapamycin and 2 μM RA stabilised mRNA expression and activity of ADH and ALDH the most closely to TSC2+/+ control levels (**Figures 3B–F**).

**Figure 4:**
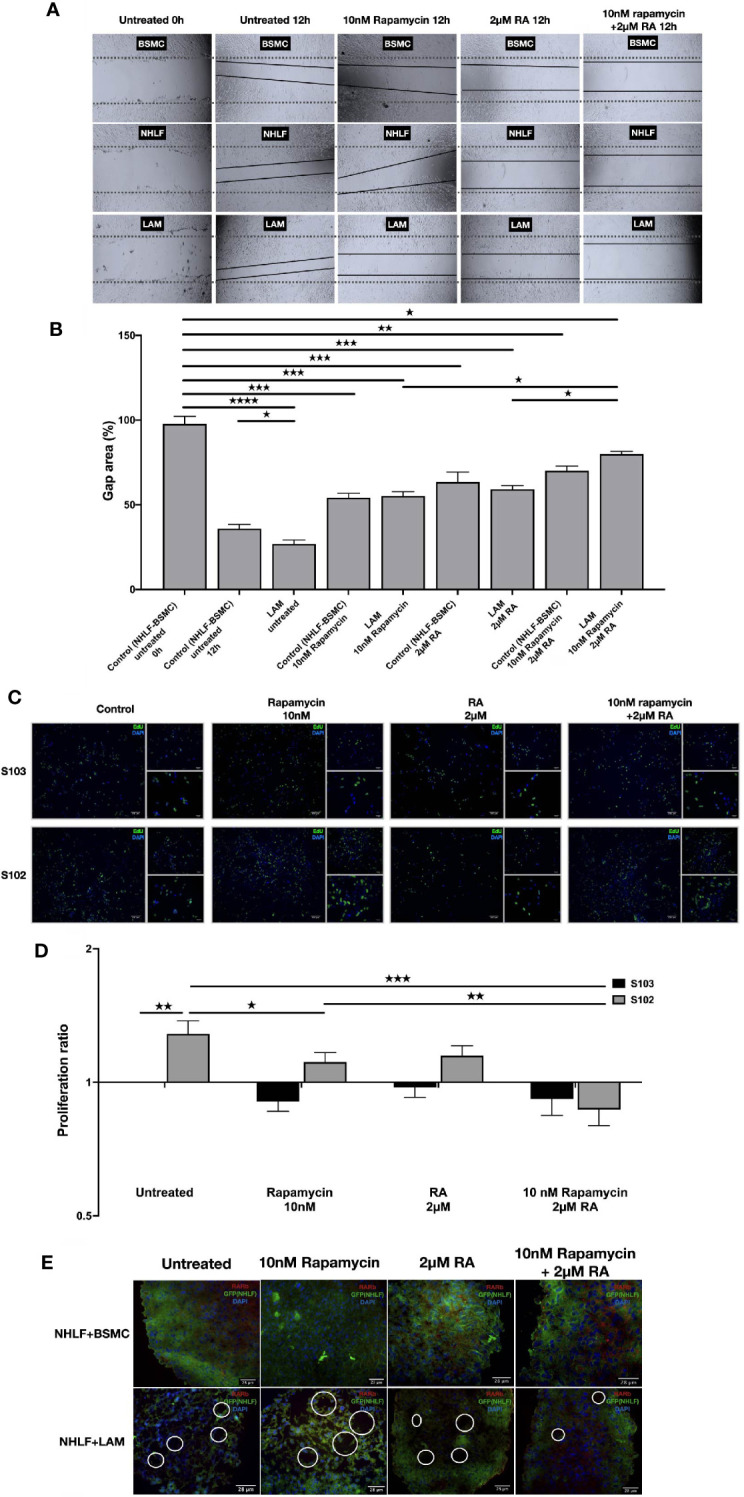
RA mono and RA and rapamycin combination treatment reduces cellular migration and proliferation in TSC-deficient cells. **(A)** Wound healing scratch assay following 10 nM rapamycin and/or 2 µM RA treatment for 24 h. **(B)** Wound gap area quantification, data are presented as gap area (%) compared to untreated control (BSMC and NHLF, 1:1) as 100%. Differences in gap closure % ± SEM. Significant changes are marked as ★, ★★, ★★★ and ★★★★ (P<0.05, P<0.001, P<0.0002 and P<0.0001, respectively). **(C, D)** Proliferation capacity of S102 compared to S103 using BrdU assay (BrdU green, DAPI blue, size-bar 100, 50, 25 μm). Proliferation ratio compared to untreated S103 ± technical error of replicates (t-test). Significant changes are marked as ★, ★★ and ★★★ (P<0.05, P<0.001 and P<0.0002 respectively). **(E)** Empty sac formation in NHLF-LAM co-cultures compared to NHLF-BSMC co-cultures in the presence or absence of 10 nM rapamycin and/or 2 µM RA treatment (24 h). Empty sac formation is marked with white circles in the staining where RARβ is red, NHLF-GFP is green and the nucleus is stained by DAPI (blue).

To test whether normalization of enzyme levels and activity in vitamin A metabolism and suppression of mTOR activity would have an effect on cellular proliferation and migration, the effect of 2 μM RA in combination with 10 nM rapamycin were tested in mono and combination treatment in a scratch and a BrdU assay (36, 37). The combined treatment of TSC2-/- cell lines with rapamycin (10 nM) and RA (2 μM) decreased cellular migration significantly (**Figures 4A, B**). Furthermore, the combination significantly decreased the proliferation capacity detected in BrdU assay compared to rapamycin mono treatment (**Figures 4C, D**).

Apart from increased cell migration and proliferation, TSC-deficiency is characterized by structural changes of the affected tissues. Such changes cannot be detected in traditional 2D cell cultures, therefore a 3D tissue aggregates were used (27). The 3D aggregate tissues containing patient derived LAM lung cell lines developed empty sac formations after 24 h incubation which feature was reduced after 10 nM rapamycin and/or 2 μM RA treatment making the LAM cell containing co-cultures structurally similar to the aggregate cultures containing only TSC+/+ BSMC and NHLF cells (**Figure 4E**). Additionally, 10 nM rapamycin and/or 2 μM RA treatments increased RARβ protein expression even in the 3D tissue structures containing TSC mutant cell lines (**S. Figure 3**).

## Discussion

In our previous study of LAM, we detected correlation between mTOR activation, mitochondrial dysfunction and downregulation of the proliferation suppressor nuclear receptor family of RAR and RXR (6). Recent studies have also shown that rapamycin treatment induced upregulation of miR-29b in LAM affected cell growth, migration, and invasion *via* regulation of RARβ activity (19).

In the present study, we confirmed that downregulation of RARβ is not just a feature of TSC-deficient cell lines (angiomyolipoma, LAM primary cell lines) but it is characteristically present in primary LAM lung tissue sections (**Figures 1D–F**). In many cancers (38) the activity of RARβ itself is suppressed *via* various pathways leading up to mTOR activation (19, 39). As RARβ levels are strongly associated with alterations in the vitamin A metabolic pathway (11), clear understanding of vitamin A metabolism in connection with TSC mutation is important for better disease control.

In the present study, we used TSC mutant angiomyolipoma and primary LAM lung derived cell lines pre-dating the rapamycin era to investigate enzyme expression and activity responsible for retinol metabolism. Based on our study the ability to metabolise retinol is seriously compromised in TSC-deficient cells (**Figure 1**). Many enzymes, including ADH1A, ADH1B, ADH1C ADH6, ALDH1A2, ALDH1A3, and ALDH3A1 were drastically upregulated, while others including ADH4, ALDH1A1, ALDH3B1, ALDH3B2, ALDH4A1 and ALDH5A1 were significantly downregulated (**Figure 1**). The characteristic function of ALDHs is to oxidize aldehydes that would otherwise participate in signalling pathways to induce cellular, to minimize ROS production and to mediate RA signalling cascades (40). Other studies have also shown that in diseases caused by TSC mutation which increases mTORC1 activity is associated with deregulation of ALDH expression and activity, resistance to oxidative stress, greater proliferation, migration, and invasion as well as higher levels of VEGF expression (32, 35). These regulatory mechanisms are important in regulation of proliferation, tumorigenesis and resistance to therapy (41). Expression of ALDHs are also regulated by RA compounds including the chemotherapeutic vitamin A or chemically related molecules (retinoids) as well as oncogenic pathways including the WNT/β-catenin and the MUC1-C/ERK pathways (40). CYP2E1 degrades retinoic acid (RA) and retinol to polar metabolites with toxic and apoptotic properties (42). Imbalance in the level of alcohol dehydrogenases acts as a competitive inhibitor of retinol oxidation in the liver which may reduce the biosynthesis of retinoic acid (43). It is especially important that co-expression and physical interactions amongst the metabolic enzymes of retinol are tightly controlled and important in regulation of cellular differentiation, proliferation, and migration.

Our experiments have highlighted that patients might benefit from combination of RA with the routinely used treatment of rapamycin in diseases affected by TSC mutation. In such treatment, reduction of rapamycin dosage and closely normalised vitamin A metabolic enzyme activities (**Figure 3**) could lead to beneficial physiological effects including reduced cellular proliferation and migration (**Figure 4**).

## Conclusion

Importantly, several enzymes of the vitamin A metabolism and the nuclear receptor RARβ can become potential therapeutic targets in TSC mutant or deregulated neoplasms (44, 45). RA for example is an FDA approved drug for acute myeloid leukaemia as RA can normalize RARβ levels and limit cancer cell migration and consequent disease progression (45). Based on our study we propose that clinical assessment of the combination of RA with reduced dosage of rapamycin might limit adverse reactions to rapamycin in rapamycin sensitive tuberous sclerosis, LAM and angiomyolipoma patients. With reduction of rapamycin levels, the inhibitory effect of rapamycin on ALDH can also become limited which allows better balance in vitamin A metabolism and consequently in RARβ activity. Increased expression and activity of RARβ might also lead to inhibition of cellular migration, proliferation and as a result improved disease control. Further studies of improved drug concentrations and clinical assessment of our *in vitro* results are certainly required to modify treatment strategies for patients suffering from diseases affected by TSC mutations.

## Data Availability Statement

The raw data supporting the conclusions of this article will be made available by the authors, without undue reservation.

## Ethics Statement

The study was approved by the Medical Research Council of Hungary (54034-4/2018/EKU). Written informed consent for participation was not required for this study in accordance with the national legislation and the institutional requirements.

## Author Contributions

EA and JB-B: performed the experiments, isolated RNA and protein from NHLF, SMC and LAM, cellular staining, embedding of samples for microscopy, performed data analysis, and prepared figures. VK: generated the LAM cell lines and performed experiments on angiomyolipoma cell lines. JM, JF, TH, and GS: selected the samples. JP designed the studies. EA and JP have written the manuscript. All authors contributed to the article and approved the submitted version.

## Funding

JP: TUDFO/51757-1/2019-ITM; 2020-4.1.1-TKP2020, GINOP 2.3.2-15-2016-00022; EFOP-3.6.1-16-2016-00004; and GINOP-2.3.3-15-2016-00012 HECRIN funds.

## Conflict of Interest Statement

The authors declare that the research was conducted in the absence of any commercial or financial relationships that could be construed as a potential conflict of interest.

## Abbreviations

ADHs/RDHs, alcohol dehydrogenases/retinol dehydrogenases; ALDHs, aldehyde dehydrogenases; ATRA, all-trans retinoic acid/tretinoin; BSMC, human bronchial smooth muscle cell; CRABP, cellular retinoic acid-binding protein; CYP, Cytochrome P450; GFP, green fluorescent protein; H&E, hematoxylin and eosin; HMB45, melanoma marker; LAM, Lymphangioleiomyomatosis; mTOR, Mammalian target of rapamycin; NHLF, normal human lung fibroblast cell; RA, Retinoic acid; RALDH, retinaldehyde dehydrogenase; RAR, Retinoic acid receptor; RBP, retinol-binding protein; RXR, retinoid x receptors; S102, human female kidney angiomyolipoma cell line 621-102 TSC2-/-; S103, human female kidney angiomyolipoma cell line 621-103 TSC2+/+; STRA6, Stimulated by retinoic acid 6; TSC 1-2 , Tuberous sclerosis 1-2.
