# Supplementary material for: Normalization of Enzyme Expression and Activity Regulating Vitamin A Metabolism Increases RAR-Beta Expression and Reduces Cellular Migration and Proliferation in Diseases Caused by Tuberous Sclerosis Gene Mutations
Source: Front Oncol. 2021 Jun 11;11:644592. doi: 10.3389/fonc.2021.644592 (PMC8226169; doi:10.3389/fonc.2021.644592)
Supplement: Supplementary file 1 [file DataSheet_1.pdf]

S. Table 1. Primer sequences

Gene name	Forward primer	Reverse primer
β-actin	GCGCGGCTACAGCTTCA	CTTAATGTCACGCACGATTTCC
TSC1	CCGTGGCCCTATGCTTGTAA	CGGCTTTGCCCACATATTCG
TSC2	CCTTGGACGGTATTGCCTGT	GCCTGCTTCTGTGTACCACT
RARβ	ATCCGAGCAGGGTTTGTCTG	TTTTCCCAGCCCCGAATCAT
ALDH1A1	GATCCCCGTGGCGTACTATG	TGGATCTTGTCAGCCCAACC
ADH1A	TCTGGGAAAAGTATCCGTACCATT	TGAAGACTGCCACAAGGGAA
ADH4	CTTTGGCCTAGGAGGTGTGG	CCAGGGCTTTAGCCTTCACA
ALDH1A2	CAAGATAGAGATGCCCGGCG	ACAGGGAACACTCTCCCACT
ALDH1A3	AGATACTTTGCAGGGTGGGC	GGGGGAAGTTCCATGGAGTG

S. Table 2. Metabolic enzymes activity in certain cancers and substrates involved

ALDH isozyme	substrate involved	Tissue	Cancer type	References
ADH 1A, B, C	Ethanol-retinol-lipid peroxidation	Liver, lung, kidneys	Oesophageal, renal, gastric	[24][25][26]
ADH 4	Ethanol-retinol	Liver, cornea	Oesophageal, renal, gastric	[24][25][26]
ADH 6	Ethanol-retinol	Liver, stomach	gastric	[24][25][26]
ALDH1A1	Retinal Aldophosphamide, Acetaldehyde	Liver, kidney, red blood cells, skeletal muscle, lung, breast, lens, stomach	All cancer types	[27]
ALDH1A2	Retinal	Testis, liver, kidney	Prostate, Acute myeloid leukaemia	[27]
ALDH1A3	Retinal	Kidney, skeletal muscle, lung, breast, stomach, salivary glands	Breast, bladder, prostate	[27]
ALDH3A1	Long-chain aliphatic aldehydes, Lipid peroxidation derived aldehydes	Stomach, cornea, breast, lung, lens, oesophagus, salivary glands, skin	Breast, lung, liver	[23]
ALDH3B1-2		Kidney, lung, pancreas	Breast, lung, colon	[23]
ALDH4A1	Pyrroline-5-carboxylate	Liver, kidney, heart, skeletal muscle, brain, pancreas, placenta, lung, spleen	Liver, glioblastoma	[28]
ALDH6A1	Methylmalonate semialdehyde	Liver, kidney, heart, skeletal muscle	Breast	[23]
CYP2E1	Catalyse the biotransformation of various compounds and enhance retinoic acid metabolism	Liver	Liver, Breast	[29]

S. Table 3. GeneMANIA report

GeneMANIA report

Application version : 3.6.0

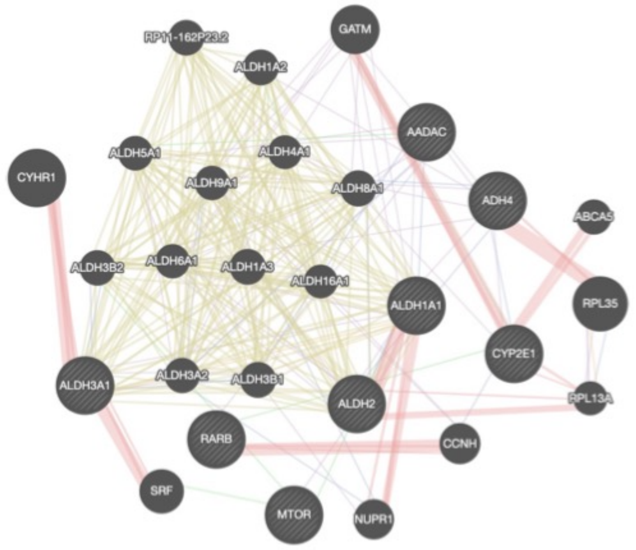

Networks

- Physical Interactions
- Shared protein domains
- Co-expression
- Co-localization
- Predicted
- Genetic Interactions

Search parameters

Organism Homo sapiens (human)

Genes RARB , ALDH2 , AADAC , CYP2E1 , ADH4 , MTOR , ALDH3 , ALDH1

Network weighting Automatically selected weighting method

Networks

A

Abu-Odeh-Aqeilan-2014 , Agrawal-Sedivy-2010 , Aichem-Groettrup-2012 , Albers-Koegl-2005 , Alexandru-Deshaies-2008 , Alizadeh-Staudt-2000 , Andresen-Flores-Morales-2014 , Arbuckle-Grant-2010 , Arroyo-Aloy-2014 , Arroyo-Aloy-2015

B

Bahr-Bowler-2013 , Bailey-Hieter-2015 , Bandyopadhyay-Ideker-2010 , Bantscheff-Drewes-2011 , Barr-Knapp-2009 , Barrios-Rodiles-Wrana-2005 , Behrends-Harper-2010 , Behzadnia-Lührmann-2007 , Bennett-Harper-2010 , Benzinger-Hermeking-2005 , Berggård-James-2006 , Bett-Hay-2013 , Bhatnagar-Attie-2014 , Bild-Nevins-2006 B , BIOGRID-SMALL-SCALE-STUDIES , BIOGRID-SMALL-SCALE-STUDIES , Blandin-Richard-2013 , Blomen-Brummelkamp-2015 , Blomen-Brummelkamp-2015 , Bogachek-Weigel-2014 , Boldrick-Relman-2002 , Bonacci-Soubeyran-2014 , Bouwmeester-Superti-Furga-2004 , Brajenovic-Drewes-2004 , Brehme-Superti-Furga-2009 , Bruderer-Hay-2011 , Burington-Shaughnessy-2008 , Butland-Hayden-2014 , Byron-Humphries-2012

C

Cai-Conaway-2007 , Camargo-Brandon-2007 , Campos-Reinberg-2015 , Cao-Chinnaiyan-2014 , Carmon-Liu-2014 , CELL_MAP , Chen-Brown-2002 , Chen-Ge-2013 , Chen-Huang-2014 , Chen-Zhang-2013 , Christianson-Kopito-2011 , Cloutier-Coulombe-2013 , Colland-Gauthier-2004 , Corominas-Iakoucheva-2014 , Couzens-Gingras-2013 , Cox-Rizzino-2013 , Coyaud-Raught-2015

D

Danielsen-Nielsen-2011 , Dart-Wells-2015 , de Hoog-Mann-2004 , Diner-Cristea-2015 , Dobbin-Giordano-2005 , Drissi-Boisvert-2015 , Dyer-Sobral-2010

E

Emanuele-Elledge-2011 , Emdal-Olsen-2015 , Ewing-Figeys-2007

F

Fenner-Prehn-2010 , Floyd-Pagliarini-2016 , Foerster-Ritter-2013 , Fogeron-Lange-2013 , Foster-Marshall-2013 , Freibaum-Taylor-2010

G

Gabriel-Baumgrass-2016 , Galligan-Howley-2015 , Gao-Reinberg-2012 , Gautier-Hall-2009 , Giannone-Liu-2010 , Glatter-Gstaiger-2009 , Gloeckner-Ueffing-2007 ,

G

Goehler-Wanker-2004 , Golebiowski-Hay-2009 , Goudreault-Gingras-2009 , Grant-2010 , Greco-Cristea-2011 , Grossmann-Stelzl-2015 , Guarani-Harper-2014 , Gupta-Pelletier-2015

H

Hanson-Clayton-2014 , Hauri-Gstaiger-2013 , Havrylov-Redowicz-2009 , Havugimana-Emili-2012 , Hayes-Urbé-2012 , Hegele-Stelzl-2012 A , Hegele-Stelzl-2012 B , Hein-Mann-2015 , Hill-Livingston-2014 , HUMANCYC , Humphries-Humphries-2009 , Hutchins-Peters-2010 , Huttlin-Gygi-2015

I

I2D-BIND-Fly2Human , I2D-BIND-Mouse2Human , I2D-BIND-Rat2Human , I2D-BIND-Worm2Human , I2D-BIND-Yeast2Human , I2D-BioGRID-Fly2Human , I2D-BioGRID-Mouse2Human , I2D-BioGRID-Rat2Human , I2D-BioGRID-Worm2Human , I2D-BioGRID-Yeast2Human , I2D-Chen-Pawson-2009-PiwiScreen-Mouse2Human , I2D-Formstecher-Daviet-2005-Embryo-Fly2Human , I2D-Giot-Rothbert-2003-Low-Fly2Human , I2D-INNATEDB-Mouse2Human , I2D-IntAct-Fly2Human , I2D-IntAct-Mouse2Human , I2D-IntAct-Rat2Human , I2D-IntAct-Worm2Human , I2D-IntAct-Yeast2Human , I2D-Krogan-Greenblatt-2006-Core-Yeast2Human , I2D-Krogan-Greenblatt-2006-NonCore-Yeast2Human , I2D-Li-Vidal-2004-CORE-1-Worm2Human , I2D-Li-Vidal-2004-non-core-Worm2Human , I2D-Manual-Mouse2Human , I2D-Manual-Rat2Human , I2D-MGI-Mouse2Human , I2D-MINT-Fly2Human , I2D-MINT-Mouse2Human , I2D-MINT-Rat2Human , I2D-MINT-Worm2Human , I2D-MINT-Yeast2Human , I2D-Ptacek-Snyder-2005-Yeast2Human , I2D-Tarassov-PCA-Yeast2Human , I2D-Tewari-Vidal-2004-TGFb-Worm2Human , I2D-vonMering-Bork-2002-High-Yeast2Human , I2D-vonMering-Bork-2002-Low-Yeast2Human , I2D-vonMering-Bork-2002-Medium-Yeast2Human , I2D-Wang-Orkin-2006-EScmplx-Mouse2Human , I2D-Wang-Orkin-2006-EScmplxlow-Mouse2Human , I2D-Yu-Vidal-2008-GoldStd-Yeast2Human , IMID , Ingham-Pawson-2005 , Innocenti-Brown-2011 , INTERPRO , IREF-BIND , IREF-BIOGRID , IREF-DIP , IREF-HPRD , IREF-INTACT , IREF-MATRIXDB , IREF-MPPI , IREF-PUBMED , IREF-SMALL-SCALE-STUDIES , IREF-SMALL-SCALE-STUDIES

J

Jeronimo-Coulombe-2007 , Jin-Pawson-2004 , Johnson-Kerner-Wichterle-2015 , Johnson-Shoemaker-2003 , Jones-MacBeath-2006 , Joshi-Cristea-2013 , Jäger-Krogan-2011

K

Kahle-Zoghbi-2011 , Kaltenbach-Hughes-2007 , Katsogiannou-Rocchi-2014 , Kim-Gygi-2011 , Kim-Major-2015 , Kneissl-Grummt-2003 , Koch-Hermeking-2007 , Kotlyar-Jurisica-2015 , Kristensen-Foster-2012 , Kärblane-Sarmiento-2015 , Kırlı-Görlich-2015

L

Lambert-Gingras-2015 , Lamoliatte-Thibault-2014 , Lau-Ronai-2012 , Lee-Songyang-2011 , Lehner-Sanderson-2004 A , Lehner-Sanderson-2004 B , Leng-Wang-2014 , Leung-Jones-2014 , Li-Chen-2015 , Li-Dorf-2011 A , Li-Dorf-2011 B , Li-Dorf-2014 , Li-Haura-2013 , Lim-Zoghbi-2006 , Lin-Smith-2010 , Lipp-Guthrie-2015 , Liu-Wang-2012 , Llères-Lamond-2010 , Loch-Strickler-2012 , Low-Heck-2014 , Lu-Zhang-2013 , Luo-Elledge-2009

M

Mak-Moffat-2010 , Mallon-McKay-2013 , Malovannaya-Qin-2010 , Markson-Sanderson-2009 , Maréchal-Zou-2014 , Matsumoto-Nakayama-2005 , McCracken-Blencowe-2005 , McFarland-Nussbaum-2008 , Meek-Piwnica-Worms-2004 , Milev-Mouland-2012 , Miyamoto-Sato-Yanagawa-2010 , Murakawa-Landthaler-2015

N

Nakayama-Ohara-2002 , Nakayasu-Adkins-2013 , Napolitano-Meroni-2011 , Narayan-Bennett-2012 , Nathan-Goldberg-2013 , NCI_NATURE , Neganova-Lako-2011 , Newman-Keating-2003 , Nicholson-Hupp-2014 , Noble-Diehl-2008

O

Oliviero-Cagney-2015 , Olma-Pintard-2009 , Oláh-Ovádi-2011 , Oshikawa-Nakayama-2012 , Ouyang-Gill-2009

P

Panigrahi-Pati-2012 , Papp-Lamia-2015 , Perez-Hernandez-Yáñez-Mó-2013 , Perou-Botstein-1999 , Perou-Botstein-2000 , Persaud-Rotin-2009 , Petschnigg-Stagljar-2014 , PFAM , Phillips-Corn-2013 , Pichlmair-Superti-Furga-2011 , Pichlmair-Superti-Furga-2012 , Pilot-Storck-Goillot-2010 , Povlsen-Choudhary-2012

R

Ramachandran-LaBaer-2004 , Raman-Harper-2015 , Ramaswamy-Golub-2001 , Ravasi-Hayashizaki-2010 , REACTOME , Reinke-Keating-2013 , Reyniers-Taymans-2014 , Richter-Chrzanowska-Lightowlers-2010 , Rieger-Chu-2004 , Rolland-Vidal-2014 , Rosenwald-Staudt-2001 , Roth-Zlotnik-2006 , Roux-Burke-2012 , Rowbotham-Mermoud-2011 , Roy-Pardo-2014 , Roy-Parent-2013 , Rual-Vidal-2005 A , Rual-Vidal-2005 B

S

Sang-Jackson-2011 , Sato-Conaway-2004 , Schadt-Shoemaker-2004 , Scholz-Taylor-2016 , Singh-Moore-2012 , Smirnov-Cheung-2009 , So-Colwill-2015 , Soler-López-Aloy-2011 , Sowa-Harper-2009 , Stehling-Lill-2012 , Stehling-Lill-2013 , Stelzl-Wanker-2005 , Stes-Gevaert-2014 , Stuart-Kim-2003 , Suter-Wanker-2013

T

Taipale-Lindquist-2012 , Taipale-Lindquist-2014 , Takahashi-Conaway-2011 , Tarallo-Weisz-2011 , Tatham-Hay-2011 , Teixeira-Gomes-2010 , Thalappilly-

T

Dusetti-2008 , Thompson-Luchansky-2014 , Tong-Moran-2014 , Toyoshima-Grandori-2012 , Tsai-Cristea-2012

U

Udeshi-Carr-2012

V

van Wijk-Timmers-2009 , Vandamme-Angrand-2011 , Varjosalo-Gstaiger-2013 , Varjosalo-Superti-Furga-2013 , Venkatesan-Vidal-2009 , Vermeulen-Mann-2010 , Vinayagam-Wanker-2011 , Virok-Fülöp-2011 , Vizeacoumar-Moffat-2013

W

Wagner-Choudhary-2011 , Wallach-Kramer-2013 , Wan-Emili-2015 , Wang-Balch-2006 , Wang-Cheung-2015 , Wang-He-2008 , Wang-Maris-2006 , Wang-Xu-2015 , Wang-Yang-2011 , Weimann-Stelzl-2013 A , Weimann-Stelzl-2013 B , Weinmann-Meister-2009 , Wen-Wu-2014 , Whisenant-Salomon-2015 , Wilker-Yaffe-2007 , Willingham-Muchowski-2003 , Witt-Labeit-2008 , Wong-O'Bryan-2012 , Woods-Monteiro-2012 , Woodsmith-Sanderson-2012 , Wu-Garvey-2007 , Wu-Li-2007 , Wu-Ma-2012 , Wu-Stein-2010 , Wu-Stein-2010

X

Xiao-Lefkowitz-2007 , Xie-Cong-2013 , Xie-Green-2012 , Xu-Ye-2012

Y

Yang-Chen-2010 , Yatim-Benkirane-2012 , Yu-Chow-2013 , Yu-Vidal-2011

Z

Zanon-Pichler-2013 , Zhang-Shang-2006 , Zhang-Zou-2011 , Zhao-Krug-2005 , Zhao-Yang-2011 , Zhou-Conrads-2004 , Zhou-Hanemann-2016

Genes

Gene	Description	Rank
AADAC	arylacetamide deacetylase [Source:HGNC Symbol;Acc:HGNC:17]	N/A
ADH4	alcohol dehydrogenase 4 (class II), pi polypeptide [Source:HGNC Symbol;Acc:HGNC:252]	N/A
ALDH2	aldehyde dehydrogenase 2 family (mitochondrial) [Source:HGNC Symbol;Acc:HGNC:404]	N/A
ALDH1A1	aldehyde dehydrogenase 1 family member A1 [Source:HGNC Symbol; Acc:HGNC:402]	N/A
RARB	retinoic acid receptor beta [Source:HGNC Symbol;Acc:HGNC:9865]	N/A
MTOR	mechanistic target of rapamycin [Source:HGNC Symbol;Acc:HGNC: 3942]	N/A
ALDH3A1	aldehyde dehydrogenase 3 family member A1 [Source:HGNC Symbol; Acc:HGNC:405]	N/A
CYP2E1	cytochrome P450 family 2 subfamily E member 1 [Source:HGNC Symbol;Acc:HGNC:2631]	N/A
CYHR1	cysteine and histidine rich 1 [Source:HGNC Symbol;Acc:HGNC:17806]	1
RPL35	ribosomal protein L35 [Source:HGNC Symbol;Acc:HGNC:10344]	2
GATM	glycine amidinotransferase [Source:HGNC Symbol;Acc:HGNC:4175]	3
SRF	serum response factor [Source:HGNC Symbol;Acc:HGNC:11291]	4
CCNH	cyclin H [Source:HGNC Symbol;Acc:HGNC:1594]	5
NUPR1	nuclear protein 1, transcriptional regulator [Source:HGNC Symbol;Acc: HGNC:29990]	6
ALDH8A1	aldehyde dehydrogenase 8 family member A1 [Source:HGNC Symbol; Acc:HGNC:15471]	7
ALDH1A3	aldehyde dehydrogenase 1 family member A3 [Source:HGNC Symbol; Acc:HGNC:409]	8
ALDH3B1	aldehyde dehydrogenase 3 family member B1 [Source:HGNC Symbol; Acc:HGNC:410]	9
ALDH1A2	aldehyde dehydrogenase 1 family member A2 [Source:HGNC Symbol; Acc:HGNC:15472]	10
RP11-162P23.2		11
ALDH3B2	aldehyde dehydrogenase 3 family member B2 [Source:HGNC Symbol; Acc:HGNC:411]	12
ALDH3A2	aldehyde dehydrogenase 3 family member A2 [Source:HGNC Symbol; Acc:HGNC:403]	13
ABCA5	ATP binding cassette subfamily A member 5 [Source:HGNC Symbol; Acc:HGNC:35]	14
ALDH5A1	aldehyde dehydrogenase 5 family member A1 [Source:HGNC Symbol; Acc:HGNC:408]	15
ALDH9A1	aldehyde dehydrogenase 9 family member A1 [Source:HGNC Symbol; Acc:HGNC:412]	16
ALDH6A1	aldehyde dehydrogenase 6 family member A1 [Source:HGNC Symbol; Acc:HGNC:7179]	17
RPL13A	ribosomal protein L13a [Source:HGNC Symbol;Acc:HGNC:10304]	18
ALDH16A1	aldehyde dehydrogenase 16 family member A1 [Source:HGNC Symbol; Acc:HGNC:28114]	19
ALDH4A1	aldehyde dehydrogenase 4 family member A1 [Source:HGNC Symbol; Acc:HGNC:406]	20

Networks

Physical Interactions 35.06%

Wang-Yang-2011 20.41%
Toward an understanding of the protein interaction network of the human liver. Wang et al (2011). *Mol Syst Biol*
Physical Interactions with 3,408 interactions from BioGRID

IREF-BIOGRID 7.76%
Physical Interactions with 155,470 interactions from iRefIndex

IREF-INTACT 6.89%
Physical Interactions with 56,297 interactions from iRefIndex

Shared protein domains 32.85%

INTERPRO 20.04%
Shared protein domains with 608,863 interactions from InterPro

PFAM 12.80%
Shared protein domains with 457,054 interactions from Pfam

Co-expression 23.82%

Mallon-McKay-2013 5.48%
StemCellDB: the human pluripotent stem cell database at the National Institutes of Health. Mallon et al (2013). *Stem Cell Res*
Co-expression with 585,265 interactions from GEO

Wu-Garvey-2007 5.41%
The effect of insulin on expression of genes and biochemical pathways in human skeletal muscle. Wu et al (2007). *Endocrine*
Co-expression with 267,109 interactions from GEO

Perou-Botstein-2000 4.61%
Molecular portraits of human breast tumours. Perou et al (2000). *Nature*
Co-expression with 185,068 interactions from supplementary material

Innocenti-Brown-2011 3.48%
Identification, replication, and functional fine-mapping of expression quantitative trait loci in primary human liver tissue. Innocenti et al (2011). *PLoS Genet*
Co-expression with 603,765 interactions from GEO

Bahr-Bowler-2013 3.35%
Peripheral blood mononuclear cell gene expression in chronic obstructive pulmonary disease. Bahr et al (2013). *Am J Respir Cell Mol Biol*
Co-expression with 274,949 interactions from GEO

Chen-Brown-2002 0.84%
Gene expression patterns in human liver cancers. Chen et al (2002). *Mol Biol Cell*
Co-expression with 282,241 interactions from supplementary material

Dobbin-Giordano-2005 0.65%
Interlaboratory comparability study of cancer gene expression analysis using oligonucleotide microarrays. Dobbin et al (2005). *Clin Cancer Res*

Co-expression 23.82%

Dobbin-Giordano-2005
Co-expression with 444,931 interactions from GEO

Co-localization 5.19%

Schadt-Shoemaker-2004 3.86%
A comprehensive transcript index of the human genome generated using microarrays and computational approaches. Schadt et al (2004). *Genome Biol*
Co-localization with 60,126 interactions from GEO

Johnson-Shoemaker-2003 1.33%
Genome-wide survey of human alternative pre-mRNA splicing with exon junction microarrays. Johnson et al (2003). *Science*
Co-localization with 426,332 interactions from GEO

Predicted 2.33%

Stuart-Kim-2003 2.33%
A gene-coexpression network for global discovery of conserved genetic modules. Stuart et al (2003). *Science*
Predicted with 24,872 interactions from supplementary material

Genetic Interactions 0.76%

Lin-Smith-2010 0.76%
A genome-wide map of human genetic interactions inferred from radiation hybrid genotypes. Lin et al (2010). *Genome Res*
Genetic Interactions with 4,820,370 interactions from supplementary material

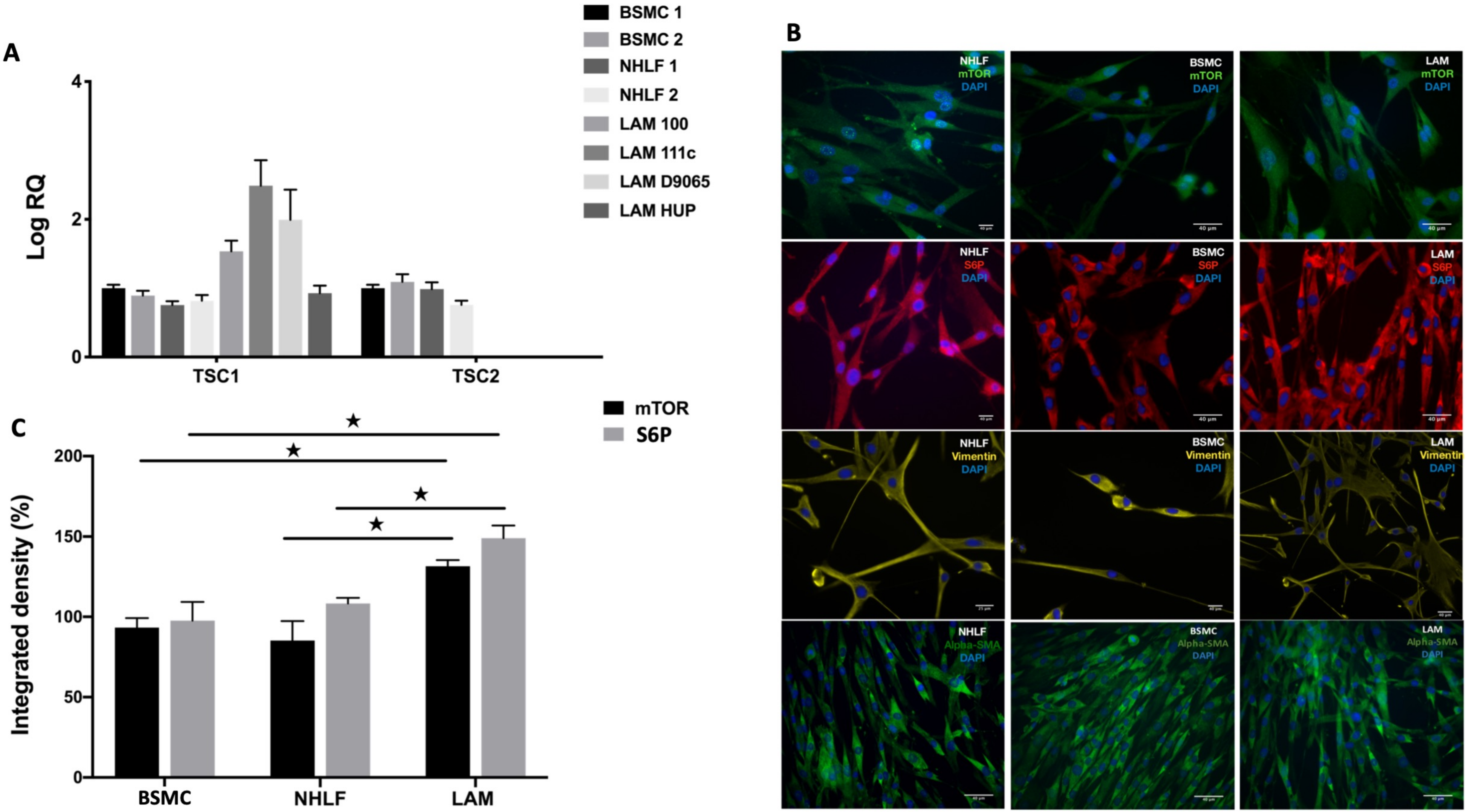

S. Figure 1. Characterizations of LAM cell lines derived from primary LAM tissues. A) loss of TSC2 in LAM cells. qRT-PCR analysis of TSC2 in LAM (n=4), NHLF (n=2) and SMC (n=2), data are presented as mean log RQ ± technical error. **B)** SMC controls (n=2), NHLF (n=2) and patient derived LAM cell lines (n=4) were stained for PS6, vimentin, mTOR and ASMA (ASMA and mTOR green, vimentin yellow, PS6 red, DAPI blue, magnification 40x, size-bar 40 μm). **C)** Quantification of fluorescent density. Data are presented as integrated density % compared to SMC±SEM, significant changes are marked as ★ (P<0.05).

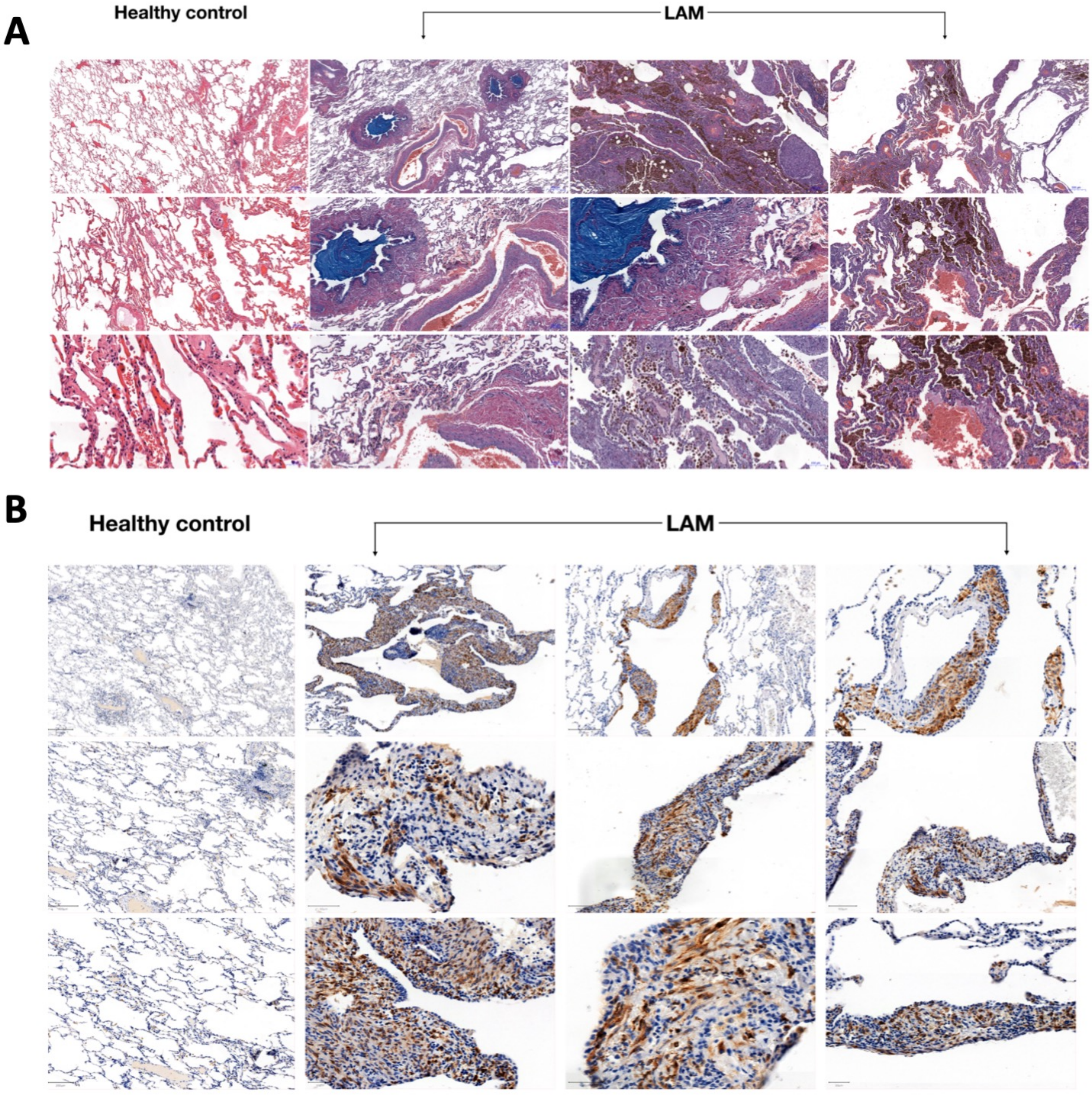

S. Figure 2. Characterizations of LAM primary LAM tissues using HE and HMB45 staining. **A)** HE staining of primary lung tissue sections using normal (n=3) and LAM (n=6) tissues. **B)** IHC of HMB45 (magnification 10x-20x size-bar 100-200 μm).

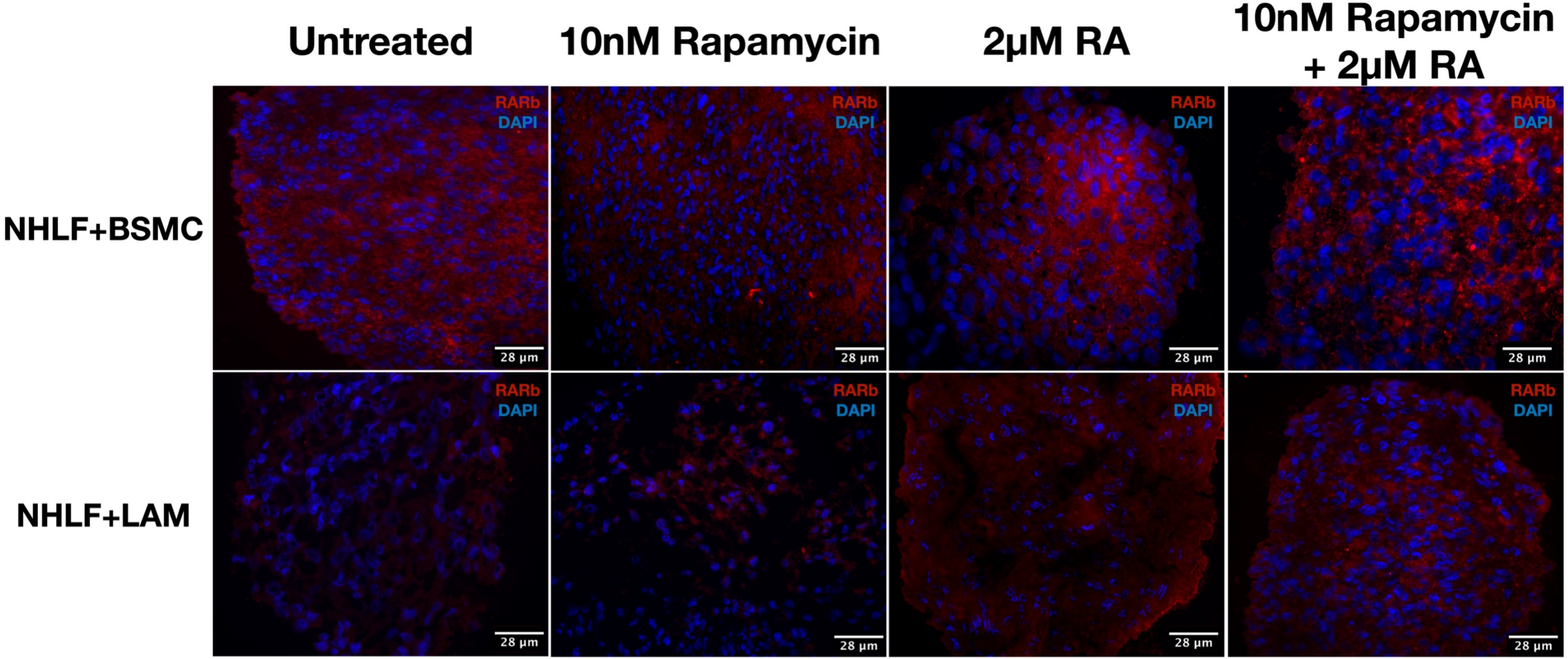

S. Figure 3. RA rescues RA expression in LAM 3D model. RARβ expression in 3D co-cultures without NHLF-GFP signal (Figure 4E) after 10 nM rapamycin and/or 2 µM RA treatment for 24 h. RARβ is red and the nucleus is stained by DAPI (blue). Magnification 40x, size-bar 28 µm.
